# Rational Engineering of Anode Current Collector for Dendrite-Free Lithium Deposition: Strategy, Application, and Perspective

**DOI:** 10.3389/fchem.2022.884308

**Published:** 2022-05-18

**Authors:** Nanrui Li, Tianqi Jia, Yanru Liu, Shifei Huang, Feiyu Kang, Yidan Cao

**Affiliations:** Institute of Materials Research & Tsinghua-Berkeley Shenzhen Institute, Shenzhen International Graduate School, Tsinghua University, Shenzhen, China

**Keywords:** lithium metal, anode current collector engineering, surface, architecture, dendrite-free

## Abstract

Lithium metal anodes have attracted extensive attention due to their high theoretical capacity and low redox potential. However, low Coulombic efficiency, serious parasitic reaction, large volume change, and dendrite growth during cycling have hindered their practical application. The engineering of an anode current collector provides important advances to solve these problems, eliminate excess lithium usage, and substantially increase the energy density. In this review, we summarize the engineering strategies of an anode current collector with emphasis on different methods and applications in lithium metal-based systems. Finally, the perspectives and challenges of current collector engineering for lithium metal anode are discussed.

## Introduction

Rapid development of electric vehicles and portable devices lead to increasing demands for high-energy density batteries. The commercial lithium-ion battery, which consists of graphite (372 mAh/g) as the anode and layered oxides as the cathode, is approaching its energy density limit and difficult to meet the growing market needs (Li et al., 2018). Lithium metal with ultrahigh theoretical capacity (3860 mAh/g), lowest redox potential (−3.04 V vs SHE), and low density (0.59 g/cm^3^) is an ideal anode candidate (Xu et al., 2014). In addition to conventional cathodes, lithium metal could also pair with lithium-free cathodes like sulfur/O_2_ to fabricate ultrahigh-energy density battery (Bruce et al., 2011).

However, the commercial application of lithium metals faces many challenges. Lithium metals react with the electrolyte immediately when in contact, forming the solid–electrolyte interphase (SEI) layer (Waldmann et al., 2018). The plating/stripping occurs on the anode current collector, which would cause unrestricted volume expansion, anode pulverization, and SEI rupture (Zhang, 2011), resulting in continuous side reactions and consumption of electrolyte/lithium. In addition, the uneven deposition of lithium leads to dendrite formation (Rosso et al., 2006; Xiao, 2019; Gao et al., 2020), which causes separator puncture, internal short circuit, and safety hazards. Dendrites may also fracture to form “dead lithium” and cause significant capacity degradation (Kolesnikov et al., 2020). Many methods, including advanced electrolyte, lithium/electrolyte interfacial engineering, and anode current collector design, have been proposed to ensure stable lithium cycling. Advanced electrolytes with novel solvent (like DX-DME (Miao et al., 2016)), lithium salts (like LiBHfip (Roy et al., 2021)), and additives (like adiponitrile (Lee et al., 2019)) have been developed to enhance Li^+^ diffusion, suppress dendrite formation, and promote stable SEI. However, the interfacial instability due to the reaction between lithium and electrolyte is still challenging, which leads to poor Coulombic efficiency (CE) and cyclability.

Cu foil is the most widely used anode current collector; however, it is not an excellent substrate for lithium deposition due to its poor lithiophilicity. The artificial interface, the composition and structure of which are goal-directed designed, has been constructed between the electrolyte and the anode to improve the interfacial stability and alleviate detrimental interfacial reactions (Guo et al., 2017). The structural design and surface engineering of the Cu current collector have also been utilized to improve its lithiophilicity, facilitate homogeneous lithium deposition, and suppress lithium dendrite formation (Cheng et al., 2021). In addition, novel current collector materials instead of Cu foil, such as carbon (Zhang Y. et al., 2017), have been investigated to fabricate low-density, low-cost, and highly-lithiophilic current collectors. Despite all the progress, it is still challenging to achieve efficient lithium plating/stripping and stable interfacial structure between the lithium metal and electrolyte.

This mini review presents the engineering strategies of anode current collectors, which includes surface engineering, architecture design, artificial SEI, and novel current collector materials, for application in different lithium metal anode–based systems. Finally, the perspectives and challenges of current collector engineering are discussed.

## Engineering Strategies for Anode Current Collector

**FIGURE 1:**
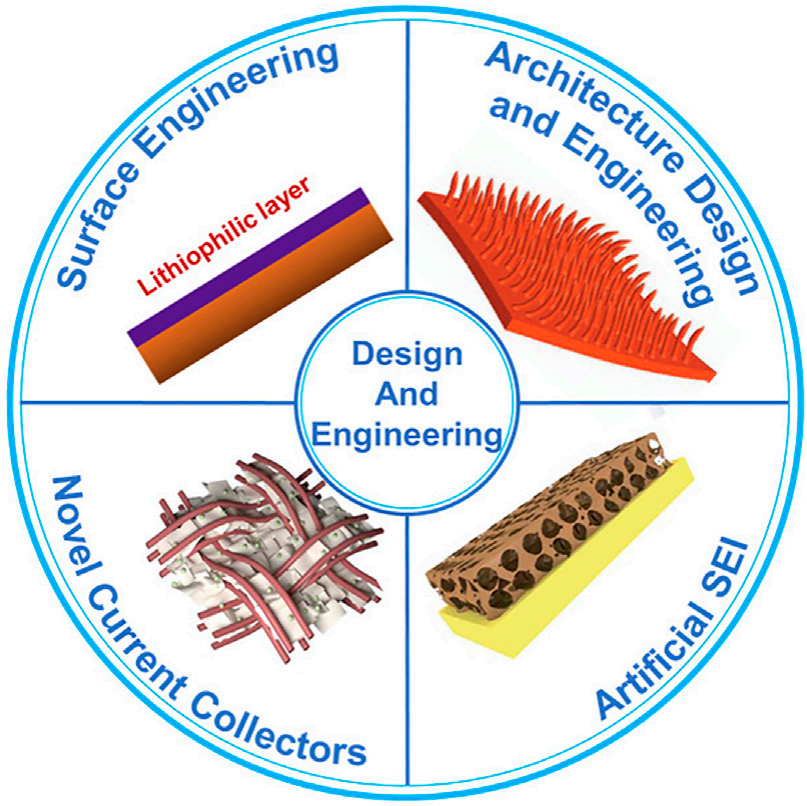
Strategies for the design and engineering of the anode current collector. Reproduced from Yang et al. (2015), Deng et al. (2018), Wu et al. (2021b) with permission from the Springer Nature, Elsevier, Royal Society of Chemistry.

The engineering of an anode current collector is decisive to achieve uniform and dendrite-free lithium deposition, since it directly affects the uniformity and morphology of lithium plating. As shown in Figure 1, there are mainly four categories of strategies: 1) surface engineering, 2) architecture design, 3) artificial SEI, and 4) novel current collector materials.

### Surface Engineering

During the plating process, the solvated lithium ions migrate from the electrolyte to the anode surface, where they are de-solvated, reduced, and heterogeneously nucleated (Li, 2007; Biswal et al., 2019). At the initial stage of deposition, one or two layers of lithium atoms are deposited, which greatly affects the subsequent deposition behavior. The structural mismatch between lithium metal and the crystal of the substrate would increase the interfacial energy, resulting in high nucleation barrier and one-dimensional dendrite growth of lithium. Researchers introduce the concept of lithiophilicity to describe the affinity to lithium of the substrate (Winand, 1991; Zhang R. et al., 2017). The nucleation overpotential, which represents the heterogeneous nucleation barrier, is directly related to the lithiophilicity of the substrate. Surface engineering of the current collector is a common method to improve its lithiophilicity, homogenize the lithium deposition, and improve cycle efficiency.

**FIGURE 2:**
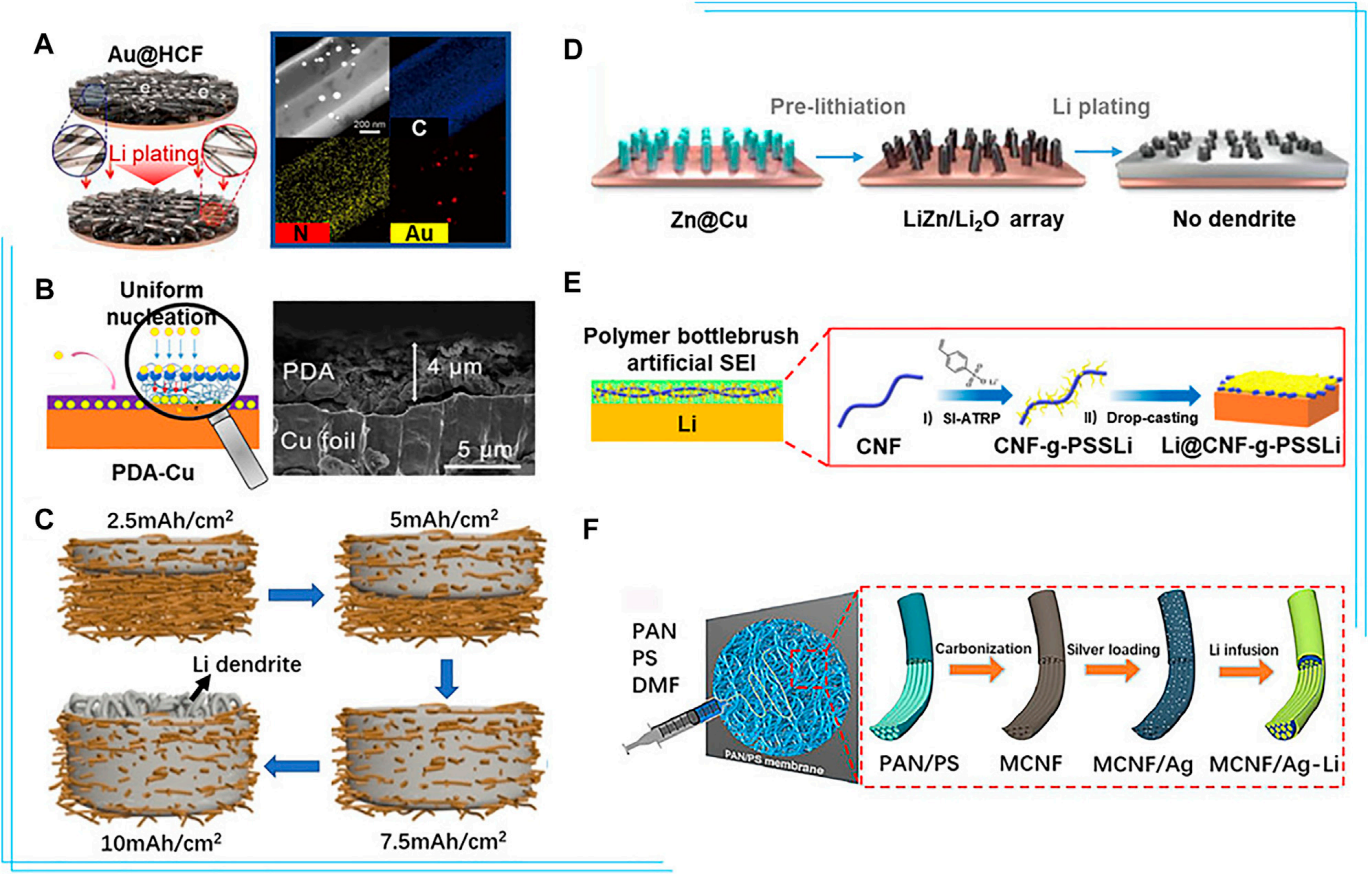
**(A)** Schematic representation of lithium plating on the bare on the Au@ hollow carbon fiber electrodes and STEM image of Au@ hollow carbon fiber and C, N, and Au EDS elemental mapping (Kim et al., 2021). **(B)** Diagram of the mechanism of polydopamine-induced Li deposition and the SEM images of the PDA-Cu foil (cross view) (He et al., 2019). **(C)** Schematic illustration of the lithium plating in the 3D Cu nanowire network current collector at different charging states (Lu et al., 2016). **(D)** Schematic illustration of lithium plating on the lithiated ZnO@Cu electrode (Wang G. et al., 2019). **(E)** Schematic illustrations of the evolution of polymer bottlebrush artificial SEI during fast charging films and the preparation of CNF-g-PSSLi (Zeng et al., 2021). **(F)** Schematic illustration of the preparation process of multichannel carbon fibers/Ag-Li composite anodes (Yu et al., 2020). Reproduced with permission from the Elsevier and American Chemical Society.

Various strategies have been employed to improve the lithiophilicity of the current collector, among which introduction of lithiophilic sites on the surface is a promising method. With studies on various metal elements, researchers have found that metals with a definite solubility in lithium, like Au and Ag, could effectively reduce the nucleation barrier and the overpotential (Li et al., 2017). The nanocapsule structure consisting of hollow carbon spheres with metal nanoparticles inside could successfully suppress dendrite formation. Hou et al. (2019) synthesized homogeneously distributed Ag nanoparticles on Cu foil *via* an electroless plating process for a lithiophilic current collector, which effectively reduced the nucleation overpotential from 240 to 50 mV, realizing uniform lithium nucleation and subsequently stable lithium plating/stripping. Kim et al. (2021) fabricated a 1D hollow carbon fiber incorporating with lithiophilic Au nanoparticles as lithiophilic nucleation sites on Cu foil (Au@HCF), reducing the current density and confining lithium to mitigate dendrite growth. The Au@HCF achieved CE of 99.9% under 1 mA/cm^2^ with 2 mAh/cm^2^ lithium plating/stripping (Figure 2A).

In addition to metals, the introduction of non-metal heteroatoms or corresponding composites is also effective to improve the lithiophilicity of the current collector. Non-metallic atoms, such as O/B/N, doped carbon is an electron-rich donor which acts as Lewis bases to attract Lewis acidic Li^+^ and induces uniform lithium deposition. Chen et al. (2019), Fang et al. (2021) reported the composite structure consisting of porous three-dimensional graphene (scaffold to suppress volumetric change) decorated with nitrogen-doped carbon nanotubes (lithiophilic sites for uniform lithium nucleation) to lead uniform lithium deposition and extend the long-term stability to 1,200 cycles at 10 mA/cm^2^. He et al. (2019) introduced a functional polydopamine (PDA) layer to the surface of Cu foil, improving CE to >97% for 100 cycles at 0.5 mA/cm^2^ (Figure 2B). Li^+^ reacted with the hydroxyl groups of the PDA layer, yielding uniformly distributed lithium complexing carbonyl groups which worked as nucleation sites and induced uniform and stable lithium plating/stripping on the current collector. Highly lithiophilic silver nanoparticles combined with polydopamine and graphene oxide have also been coated on Cu to facilitate stable lithium cycling (Wondimkun et al., 2021). In addition, other polymers like PVDF (Gao et al., 2019), PMMA (Zhou et al., 2020), and PEO (Assegie et al., 2018) have also been developed to coat Cu foil.

Surface engineering is one of the most widely studied methods for current collector engineering. Various materials, including lithiophilic elements, metal alloys, and polymers, and synthesis methods have been explored to effectively improve the lithiophilicity of the anode current collector. Despite these progresses, there are still many challenges for practical application. The working mechanism of the surface modifications, especially for polymer and composite materials, is still lacking. The evolution of the surface modification during cycling has not been well understood. In addition, most of the reported surface coatings are liable to be destroyed or pulverized. It is still challenging for current collector engineering to achieve stable surface coating with uniform lithiophilic sites as well as alleviate anode/electrolyte side reactions.

### Architecture Design

Although surface lithiophilic modification to induce uniform lithium deposition could stabilize lithium cycling to some extent, it cannot deal with the volume change and pulverization of Li during plating/stripping. Lowering the local current density along the anode surface with a 3D porous structured current collector could alleviate uneven Li^+^ flux (Li et al., 2016), retard the onset of dendrite nuclei, and reduce the dendrite growth (Xu et al., 2014; Yang et al., 2015). Meanwhile, the architecture could facilitate Li + migration and undermine the volume change of lithium (Ni et al., 2020).

Lu et al. (2016) designed a free-standing Cu nanowire network to accommodate lithium and limit dendrites/expansion (Figure 2C), resulting in enhanced cycling stability with average CE of 98.6% over 200 cycles and outstanding rate performance owing to the high conductivity of the network. Wang L.-M. et al. (2019) fabricated a Cu current collector consisting of finger-like pores as microchannels for electrolyte and Li^+^ diffusion, and random small pores as cages to accommodate lithium deposits. The CE of Li||Cu cell maintains 97.6% for 200 cycles. Introducing the lithiophilic components into the 3D structure could further enhance uniform lithium plating/stripping, prevent dendrite formation, and improve the cycling. Synthesis of a lithiophilic 3D porous Cu–Zn current collector with a lithiophilic residual Cu–Zn alloy by chemical dealloying brass has been reported (Zhang D. et al., 2019). Similarly, Cu decorated with the aligned LiZn/Li_2_O nanorod arrays (Figure 2D) was used to regulate the lithium plating/stripping behavior, reduce the local current density, and alleviate the volume change during cycling. With the suppression of dendrites growth on the current collector, it exhibits high CE up to 93.3% after 200 cycles (Wang G. et al., 2019). Hierarchically porous Cu with various types of lithiophilic components, including Li-Sb interphase (Wang J. et al., 2021) and Cu_x_O (Wang Y. et al., 2021) have been reported to simultaneously lower the local current density and reduce the lithium nucleation barrier.

Lithium preferentially deposits on top of the 3D structure, which easily causes blockage and short-circuit. Novel gradient structure has been developed to induce homogeneous instead of top deposition. Zheng et al. (2020) fabricated a 3D porous lithiophilic–lithiophobic–lithiophilic dual-gradient Cu-Au-ZnO-PAN-ZnO (CAZPZ) current collector. The Au and ZnO at the bottom were more lithiophilic, while the ZnO-PAN-ZnO skeleton provided space to accommodate deposited lithium and the lithiated ZnO (Li_2_O/Li_x_Zn) layer regulated Li^+^ flux. As a result, long-term stabilization for 1200 h at 0.5 mA/cm^2^ and a low overpotential of 22 mV at 3 mA/cm^2^ were achieved in symmetric cells. 3D Si@carbon nanofibers (CNFs)@ZnO-ZnO-Cu skeleton (SCZ) (Zhang et al., 2021) for homogeneous bottom-growth of lithium was reported, in which the top-growth of lithium was successfully avoided by the conductivity and overpotential gradient induced by the top Li_x_Si@CNF and bottom Li_y_Zn@CNF layers.

Internal resistance and electroactive surface area increase often accompany with the 3D structures, which may reduce the effective current density, and facilitate electrolyte decomposition at the anode/electrolyte interface. The combination of architecture design with other strategies, like surface engineering and artificial SEI, is a hot topic in the investigation for anode current collector engineering. However, the complicated manufacturing process of these nanostructured materials restricts their practical application. The mechanical stability of the designed architecture is another concern regarding to practical application. It is essential to develop facile strategies for the fabrication of anode current collector with carefully designed architectures, which could simultaneously achieve excellent electrochemical performance, cycling stability and cost effectiveness.

### Artificial Solid–Electrolyte Interphase

SEI is a Li^+^ conductive and electron insulative interphase passivation layer forming on the surface of the electrode material by interfacial reaction between the electrode and electrolyte during initial cycle, and its stability greatly affects the batteries’ cyclability. The fragility of SEI and deformability of Li would cause SEI rupture and continuous consumption of electrolyte and lithium due to lithium/electrolyte side reactions, impeding the long-term cycling. Hence, the strategy of constructing stable artificial SEI is proposed to suppress the growth of lithium dendrites and improve cyclability (Peled and Menkin, 2017; Shi et al., 2018).

Various materials have been used to establish artificial SEI and facilitate stable Li plating/stripping. Inorganic materials such as metal chloride perovskite and carbon can shield Li from liquid electrolyte and allow fast Li^+^ shuttle (Yin et al., 2020). For example, Wu Q. et al. (2021) proposed an oxygen defect-rich carbon with MgO_x_ domains as a 3D monolithic host and artificial SEI film simultaneously. However, inorganic artificial SEI is prone to fracture due to volume variation of lithium. Polymers with superior flexibility and high elasticity well offset this disadvantage (Ma et al., 2020). However, polymer-based artificial SEI is difficult to inhibit the dendrite growth during long-term cycling due to its low mechanical modulus. Therefore, organic–inorganic composite materials are used for artificial SEI. Zhong et al. (2019) fabricated a lithium alginate-based artificial SEI layer that allows fast Li^+^ transport and stable operation. Zeng et al. (2021) proposed a superstructured single-ion conducting polymer brush (CNF-g-PSSLi) as artificial SEI layer (Figure 2E), which contained robust cellulose nanofibril (CNF) backbone nanonetwork to ensure excellent mechanical properties with 5.3 GPa elasticity modulus. Poly (lithium *p*-styrenesulfonate) (PSSLi) side chains with rich -SO_3_^−^ functional groups coordinating with lithium ions provided fast ion transport channels to enable excellent rate performance and avoid undesirable detachment.

Artificial SEI is an ingenious method for anode current collector engineering, which can purposefully manipulate the interfacial composition and stability. Organic–inorganic composite materials as artificial SEI films effectively address the limitations of organic polymer or inorganic SEI films. However, appropriate methods to incorporate inorganic components into polymer and develop multifunctional SEI are still highly desired. With much attention on the artificial SEI study, constructing artificial SEI films with high mechanical stability, good elasticity, excellent electrochemical compatibility, and high current density endurance is still urgently needed for anode current collector engineering.

### Novel Current Collectors

The traditional copper current collector is lithiophobic, which easily induces inhomogeneous lithium deposition, dendrite formation, and poor lithium cycling efficiency (Pande and Viswanathan, 2019). Novel materials, including carbon-based and polymer-based materials, have emerged for the fabrication of anode current collector to overcome the defect of traditional Cu.

Carbon-based material exhibits great promise as alternative current collector for lithium metal due to its excellent mechanical strength, light weight, easily-manipulated architecture, and excellent chemical stability. Matsuda et al. (2017) fabricated the free-standing polyacrylonitrile-based insulative microfiber matrix to enable stable Li cycling at 10 mAh/cm^2^. In addition, the lithiophilic modification of carbon-based current collector is also important. Deng et al. (2018) found carbon fiber cloth, which confined lithium in micro-channels, improved the reversibility of lithium. Furthermore, graphene sheets in micro-channels and lithiophilic ZnO nanoparticles on fibers were constructed to simultaneously provide powerful conductive network, abundant deposition sites and strong surface lithiophilicity. Inspired by the structure of the lotus root, Yu et al. (2020) reported 3D multichannel carbon fibers decorated with lithiophilic Ag nanoparticles (Figure 2F). The produced multi-channels provided sufficient space for volume changes of lithium, while Ag nanoparticles ensured homogeneous nucleation and deposition. Kwon et al. (2021) synthesized an atomically defective carbon current collector with multivacancy defects using the surface-oxidized carbon fiber paper to induce homogeneous SEI formation and uniform lithium growth. Polymers usually contain a number of polar groups, such as hydroxyl, nitro, and carboxyl, which possess excellent lithiophilicity. Therefore, the polymer materials are also promising as novel current collector. However, polymer is hardly to be used alone. Wang et al. (2018) used a 3D polyvinyl nanofiber network with sufficient polar functional groups (O-H, C-H, C-O) to reinforce Li^+^–polymer interaction and regulate the uniform lithium deposition in the voids between the nanofibers. Ju et al. (2020) made a biomacromolecule matrix by trifluoroethanol-modified natural eggshell membrane to control lithium growth and achieve an ultralong cycling life of over 1200 h at 5 mA/cm^2^. Weldeyohannes et al. (2021) designed the gold sputter perforated polyimide film (PI@Au) as the anode current collector to guide the lithium plating/stripping with an average CE of 98.7%.

Novel carbon-based and polymer-based materials are attracting increasing attention on the development of current collectors for lithium anode due to their light weight, flexibility, and accessibility for structural/compositional regulation. Despite these studies, the electronic conductivity, electrochemical performance, mechanical strength, and stability of polymer- or carbon-based materials are still not satisfying to fabricate free-standing current collectors. In addition, low-cost fabrication approaches which are comparable to Cu foil are also essential.

## Application of Current Collector Engineering

Anode current collector engineering has been widely used in various systems, including anode-free lithium metal battery (AFLMB), all-solid-state Li metal battery (ASSB), and lithium–sulfur battery (LSB).

For AFLMB, the Li^+^ from the cathode is directly plated on anode current collector; however, the cycling performance is quite poor due to the low CE (Qian et al., 2016). Current collector engineering is an effective method to improve cyclability and replenish lithium in AFLMB. Benzotriazole (BTA) with lithiophilic N atoms was used on Cu to guide homogeneous Li^+^ plating/stripping (Kang et al., 2020). The capacity retention of the anode-free cell was improved from 13.3% to ∼73.3% after 50 cycles. The atomically defective carbon current collector invented by Kwon et al. (2021) successfully elevated the capacity retention of AFLMB to 90% over 50 cycles under lean electrolyte conditions. Lin et al. (2021) applied an epitaxial induced plating current collector (E-Cu) with a GaInSn liquid metal (LM) layer to facilitate Li^+^ diffusion and initiate epitaxial growth of lithium, resulting in increased capacity retention from 66% to 84% in 50 cycles of an anode-free NCM811||E-Cu pouch cell with a remarkable energy density of 420 Wh/kg.

ASSB applies a solid electrolyte and lithium metal anode to achieve high energy density (>500 Wh/kg) and power density (>10 kw/kg) (Pervez et al., 2019). However, ASSB exhibits high anode/electrolyte interfacial resistance, the uneven distribution of interfacial current accelerates the dendrite growth, and anode current collector engineering is an efficient approach to solve these problems. Wu M. et al. (2021) introduced a graphene and periodic wrinkled structure on the surface of Cu by CVD to improve adhesion, reduce interfacial resistance, and improve the reversibility. Au- or Ni-coated current collectors with microsized pores, which facilitate uniform current distribution, were reported to deposit Li in the engineered pores and obtain stable lithium plating/stripping in ASSB (Shinzo et al., 2020).

Lithium–sulfur (Li-S) battery is another promising high-energy density battery utilizing a lithium anode paired with a sulfur cathode. However, the intermediate discharge products, that is, polysulfides, dissolve into the electrolyte, migrate between the anode and cathode, and react with the anode, destroying the SEI and causing current collector corrosion. Therefore, polysulfide corrosion-resistant and lithium deposition-friendly anode current collector is highly desired. Urbonaite et al. (2015), Zhang X. et al. (2019) proposed a lightweight corrosion-resistant 3D Ti current collector, which not only mitigated the incompatibility between polysulfides and current collectors but also ensured uniform lithium cycling. As a result, CE of 99.1% over 1000 h at high deposition capacity of 5 mAh/cm^2^ without dendrites was achieved. Jin et al. (2016) proposed a 3D current collector composed of micrometer-long carbon (CNT) bonded to an ultrathin graphite foam (NGF) as both anode and cathode current collectors. The Li-S battery consisting of a S/CNT-NGF cathode and a Li/CNT-NGF anode exhibited excellent cycling stability for 4,000 cycles with 0.057% capacity decay per cycle.

## Summary, Challenges, and Perspectives

In summary, recent progress of the rational design and engineering of anode current collector and their applications in various lithium metal-based batteries are reviewed in this study. There are mainly four methods, namely, surface engineering, architecture design, artificial SEI, and alternative current collectors, to address the main issues regarding to the unstable lithium–electrolyte interface and uncontrolled lithium growth on the anode current collector. Although the current collector engineering could effectively improve the CE and cycle life of the lithium metal, further improvement of electrochemical performance regarding the following aspects and practical considerations, like compatibility with electrolyte, cost, and fabrication efficiency, still remain challenging.

1) *Lithiophilicity* of the current collector is a key to alleviate the dendrite formation. More efficient materials and synthesis methods to achieve the accommodating surface modification layer are needed. Surface engineering at the atomic level to effectively reduce the weight of the modification layer and improve the energy density and lithiophilicity is essential. 2) The *3D structure and porous structure* of substrates, resulting in a high specific surface area, may increase the side reactions with the electrolyte. Alleviating side reactions to reduce the lithium consumption and increasing the stability of special structures are highly desired. The combination of a 3D microstructure and lithiophilic surface coating would be an important direction of the future research. 3) *Artificial SEI* design methodology, including compatibility with electrolyte, interfacial interaction effects on performance, and material composition manipulation, needs to be established based on expanded knowledge on the composition and function of the SEI in different cases. With deeper understanding on the methodology, development of multifunctional artificial SEI is quite promising in the future. 4) *Novel current collectors* with low cost, light weight, flexibility, and high lithiophilicity are attractive and promising. However, the mechanical strength, electronic conductivity, and compatibility of novel materials need to be enhanced by using low-cost fabrication approaches before practical application. Limited investigations have been reported, and exploration on alternative novel materials for anode current collectors and corresponding fabrication methods would lead to further performance improvement of lithium metal anodes.

## References

[B1] Assegie A. A. Cheng J.-H. Kuo L.-M. Su W.-N. Hwang B.-J. (2018). Polyethylene Oxide Film Coating Enhances Lithium Cycling Efficiency of an Anode-free Lithium-Metal Battery. Nanoscale 10, 6125–6138. 10.1039/c7nr09058g 29557449

[B2] Biswal P. Stalin S. Kludze A. Choudhury S. Archer L. A. (2019). Nucleation and Early Stage Growth of Li Electrodeposits. Nano Lett. 19, 8191–8200. 10.1021/acs.nanolett.9b03548 31566985

[B3] Bruce P. G. Freunberger S. A. Hardwick L. J. Tarascon J.-M. (2011). Li-O2 and Li-S Batteries with High Energy Storage. Nat. Mater 11, 19–29. 10.1038/nmat3191 22169914

[B4] Chen X. Chen X. R. Hou T. Z. Li B. Q. Cheng X. B. Zhang R. (2019). Lithiophilicity Chemistry of Heteroatom-Doped Carbon to Guide Uniform Lithium Nucleation in Lithium Metal Anodes. Sci. Adv. 5, eaau7728. 10.1126/sciadv.aau7728 30793031 PMC6377277

[B5] Cheng Y. Chen J. Chen Y. Ke X. Li J. Yang Y. (2021). Lithium Host:Advanced Architecture Components for Lithium Metal Anode. Energy Storage Mater. 38, 276–298. 10.1016/j.ensm.2021.03.008

[B6] Deng W. Zhu W. Zhou X. Liu Z. (2018). Graphene Nested Porous Carbon Current Collector for Lithium Metal Anode with Ultrahigh Areal Capacity. Energy Storage Mater. 15, 266–273. 10.1016/j.ensm.2018.05.005

[B7] Fang Y. Hsieh Y.-Y. Khosravifar M. Johnson K. Kwasi Adusei P. Kanakaraj S. N. (2021). Lithiophilic Current Collector Based on Nitrogen Doped Carbon Nanotubes and Three-Dimensional Graphene for Long-Life Lithium Metal Batteries. Mater. Sci. Eng. B 267, 115067. 10.1016/j.mseb.2021.115067

[B8] Gao X. Zhou Y.-N. Han D. Zhou J. Zhou D. Tang W. (2020). Thermodynamic Understanding of Li-Dendrite Formation. Joule 4, 1864–1879. 10.1016/j.joule.2020.06.016

[B9] Gao Z. Zhang S. Huang Z. Lu Y. Wang W. Wang K. (2019). Protection of Li Metal Anode by Surface-Coating of PVDF Thin Film to Enhance the Cycling Performance of Li Batteries. Chin. Chem. Lett. 30, 525–528. 10.1016/j.cclet.2018.05.016

[B10] Guo Y. Li H. Zhai T. (2017). Reviving Lithium-Metal Anodes for Next-Generation High-Energy Batteries. Adv. Mater 29, 1700007. 10.1002/adma.201700007 28585291

[B11] He Y. Xu H. Shi J. Liu P. Tian Z. Dong N. (2019). Polydopamine Coating Layer Modified Current Collector for Dendrite-Free Li Metal Anode. Energy Storage Mater. 23, 418–426. 10.1016/j.ensm.2019.04.026

[B12] Hou Z. Yu Y. Wang W. Zhao X. Di Q. Chen Q. (2019). Lithiophilic Ag Nanoparticle Layer on Cu Current Collector toward Stable Li Metal Anode. ACS Appl. Mat. Interfaces 11, 8148–8154. 10.1021/acsami.9b01521 30707016

[B13] Jin S. Xin S. Wang L. Du Z. Cao L. Chen J. (2016). Covalently Connected Carbon Nanostructures for Current Collectors in Both the Cathode and Anode of Li-S Batteries. Adv. Mat. 28, 9094–9102. 10.1002/adma.201602704 27604953

[B14] Ju Z. Nai J. Wang Y. Liu T. Zheng J. Yuan H. (2020). Biomacromolecules Enabled Dendrite-Free Lithium Metal Battery and its Origin Revealed by Cryo-Electron Microscopy. Nat. Commun. 11, 488. 10.1038/s41467-020-14358-1 31980618 PMC6981142

[B15] Kang T. Zhao J. Guo F. Zheng L. Mao Y. Wang C. (2020). Dendrite-Free Lithium Anodes Enabled by a Commonly Used Copper Antirusting Agent. ACS Appl. Mat. Interfaces 12, 8168–8175. 10.1021/acsami.9b19655 31986006

[B16] Kim B. G. Kang D. W. Park G. Park S. H. Lee S.-M. Choi J. W. (2021). Electrospun Li-Confinable Hollow Carbon Fibers for Highly Stable Li-Metal Batteries. Chem. Eng. J. 422, 130017. 10.1016/j.cej.2021.130017

[B17] Kolesnikov A. Kolek M. Dohmann J. F. Horsthemke F. Börner M. Bieker P. (2020). Galvanic Corrosion of Lithium‐Powder‐Based Electrodes. Adv. Energy Mater. 10, 2000017. 10.1002/aenm.202000017

[B18] Kwon H. Lee J.-H. Roh Y. Baek J. Shin D. J. Yoon J. K. (2021). An Electron-Deficient Carbon Current Collector for Anode-Free Li-Metal Batteries. Nat. Commun. 12, 5537. 10.1038/s41467-021-25848-1 34545077 PMC8452779

[B19] Lee S. H. Hwang J. Y. Park S. J. Park G. T. Sun Y. K. (2019). Adiponitrile (C_6_H_8_N_2_): A New Bi‐Functional Additive for High‐Performance Li‐Metal Batteries. Adv. Funct. Mater. 29, 1902496. 10.1002/adfm.201902496

[B20] Li J. (2007). The Mechanics and Physics of Defect Nucleation (Vol 32, Pg 151, 2007). Mrs Bull. 32, 462. 10.1557/mrs2007.48

[B21] Li M. Lu J. Chen Z. Amine K. (2018). 30 Years of Lithium-Ion Batteries. Adv. Mater 30, e1800561. 10.1002/adma.201800561 29904941

[B22] Li N.-W. Yin Y.-X. Yang C.-P. Guo Y.-G. (2016). An Artificial Solid Electrolyte Interphase Layer for Stable Lithium Metal Anodes. Adv. Mat. 28, 1853–1858. 10.1002/adma.201504526 26698171

[B23] Li Q. Zhu S. Lu Y. (2017). 3D Porous Cu Current Collector/Li-Metal Composite Anode for Stable Lithium-Metal Batteries. Adv. Funct. Mater. 27, 1606422. 10.1002/adfm.201606422

[B24] Lin L. Suo L. Hu Y. S. Li H. Huang X. Chen L. (2021). Epitaxial Induced Plating Current‐Collector Lasting Lifespan of Anode‐Free Lithium Metal Battery. Adv. Energy Mater. 11, 2003709. 10.1002/aenm.202003709

[B25] Lu L.-L. Ge J. Yang J.-N. Chen S.-M. Yao H.-B. Zhou F. (2016). Free-Standing Copper Nanowire Network Current Collector for Improving Lithium Anode Performance. Nano Lett. 16, 4431–4437. 10.1021/acs.nanolett.6b01581 27253417

[B26] Ma L. Cui J. Yao S. Liu X. Luo Y. Shen X. (2020). Dendrite-Free Lithium Metal and Sodium Metal Batteries. Energy Storage Mater. 27, 522–554. 10.1016/j.ensm.2019.12.014

[B27] Matsuda S. Kubo Y. Uosaki K. Nakanishi S. (2017). Insulative Microfiber 3D Matrix as a Host Material Minimizing Volume Change of the Anode of Li Metal Batteries. ACS Energy Lett. 2, 924–929. 10.1021/acsenergylett.7b00149

[B28] Miao R. Yang J. Xu Z. Wang J. Nuli Y. Sun L. (2016). A New Ether-Based Electrolyte for Dendrite-free Lithium-Metal Based Rechargeable Batteries. Sci. Rep. 6, 21771. 10.1038/srep21771 26878890 PMC4754942

[B29] Ni S. Tan S. An Q. Mai L. (2020). Three Dimensional Porous Frameworks for Lithium Dendrite Suppression. J. Energy Chem. 44, 73–89. 10.1016/j.jechem.2019.09.031

[B30] Pande V. Viswanathan V. (2019). Computational Screening of Current Collectors for Enabling Anode-free Lithium Metal Batteries. ACS Energy Lett. 4, 2952–2959. 10.1021/acsenergylett.9b02306

[B31] Peled E. Menkin S. (2017). Review-SEI: Past, Present and Future. J. Electrochem. Soc. 164, A1703–A1719. 10.1149/2.1441707jes

[B32] Pervez S. A. Cambaz M. A. Thangadurai V. Fichtner M. (2019). Interface in Solid-State Lithium Battery: Challenges, Progress, and Outlook. ACS Appl. Mat. Interfaces 11, 22029–22050. 10.1021/acsami.9b02675 31144798

[B33] Qian J. Adams B. D. Zheng J. Xu W. Henderson W. A. Wang J. (2016). Anode-Free Rechargeable Lithium Metal Batteries. Adv. Funct. Mat. 26, 7094–7102. 10.1002/adfm.201602353

[B34] Rosso M. Brissot C. Teyssot A. Dollé M. Sannier L. Tarascon J.-M. (2006). Dendrite Short-Circuit and Fuse Effect on Li/Polymer/Li Cells. Electrochimica Acta 51, 5334–5340. 10.1016/j.electacta.2006.02.004

[B35] Roy B. Cherepanov P. Nguyen C. Forsyth C. Pal U. Mendes T. C. (2021). Lithium Borate Ester Salts for Electrolyte Application in Next‐Generation High Voltage Lithium Batteries. Adv. Energy Mater. 11, 2101422. 10.1002/aenm.202101422

[B36] Shi F. Pei A. Boyle D. T. Xie J. Yu X. Zhang X. (2018). Lithium Metal Stripping beneath the Solid Electrolyte Interphase. Proc. Natl. Acad. Sci. U.S.A. 115, 8529–8534. 10.1073/pnas.1806878115 30082382 PMC6112724

[B37] Shinzo S. Higuchi E. Chiku M. Hayashi A. Inoue H. (2020). Control of Dendritic Growth of the Lithium Metal in All-Solid-State Lithium Metal Batteries: Effect of the Current Collector with Microsized Pores. ACS Appl. Mat. Interfaces 12, 22798–22803. 10.1021/acsami.0c01759 32281781

[B38] Urbonaite S. Poux T. Novák P. (2015). Progress towards Commercially Viable Li-S Battery Cells. Adv. Energy Mater. 5, 1500118. 10.1002/aenm.201500118

[B39] Waldmann T. Hogg B.-I. Wohlfahrt-Mehrens M. (2018). Li Plating as Unwanted Side Reaction in Commercial Li-Ion Cells - A Review. J. Power Sources 384, 107–124. 10.1016/j.jpowsour.2018.02.063

[B40] Wang G. Xiong X. Lin Z. Zheng J. Fenghua Z. Li Y. (2018). Uniform Li Deposition Regulated via Three-Dimensional Polyvinyl Alcohol Nanofiber Networks for Effective Li Metal Anodes. Nanoscale 10, 10018–10024. 10.1039/c8nr01995a 29774917

[B41] Wang G. Xiong X. Zou P. Fu X. Lin Z. Li Y. (2019a). Lithiated Zinc Oxide Nanorod Arrays on Copper Current Collectors for Robust Li Metal Anodes. Chem. Eng. J. 378, 122243. 10.1016/j.cej.2019.122243

[B42] Wang J. Wang M. Chen F. Li Y. Zhang L. Zhao Y. (2021a). In-situ Construction of Lithiophilic Interphase in Vertical Micro-channels of 3D Copper Current Collector for High Performance Lithium-Metal Batteries. Energy Storage Mater. 34, 22–27. 10.1016/j.ensm.2020.09.002

[B43] Wang L.-M. Tang Z.-F. Lin J. He X.-D. Chen C.-S. Chen C.-H. (2019b). A 3D Cu Current Collector with a Biporous Structure Derived by a Phase Inversion Tape Casting Method for Stable Li Metal Anodes. J. Mat. Chem. A 7, 17376–17385. 10.1039/c9ta05357c

[B44] Wang Y. Zhao Z. Zeng W. Liu X. Wang L. Zhu J. (2021b). Hierarchically Porous Cu Current Collector with Lithiophilic Cu O Interphase towards High-Performance Lithium Metal Batteries. J. Energy Chem. 58, 292–299. 10.1016/j.jechem.2020.10.005

[B45] Weldeyohannes H. H. Abrha L. H. Nikodimos Y. Shitaw K. N. Hagos T. M. Huang C.-J. (2021). Guiding Lithium-Ion Flux to Avoid Cell's Short Circuit and Extend Cycle Life for an Anode-Free Lithium Metal Battery. J. Power Sources 506, 230204. 10.1016/j.jpowsour.2021.230204

[B46] Winand R. (1991). Electrocrystallization: Fundamental Considerations and Application to High Current Density Continuous Steel Sheet Plating. J. Appl. Electrochem 21, 377–385. 10.1007/bf01024572

[B47] Wondimkun Z. T. Tegegne W. A. Shi-Kai J. Huang C.-J. Sahalie N. A. Weret M. A. (2021). Highly-Lithiophilic Ag@PDA-GO Film to Suppress Dendrite Formation on Cu Substrate in Anode-Free Lithium Metal Batteries. Energy Storage Mater. 35, 334–344. 10.1016/j.ensm.2020.11.023

[B48] Wu M. Kim J. Y. Chae O. B. Jung W.-B. Choi S. Kim D. Y. (2021a). Nanoscale Wrinkled Cu as a Current Collector for High-Loading Graphite Anode in Solid-State Lithium Batteries. ACS Appl. Mat. Interfaces 13, 2576–2583. 10.1021/acsami.0c04769 33400505

[B49] Wu Q. Yao Z. Du A. Wu H. Huang M. Xu J. (2021b). Oxygen-Defect-Rich Coating with Nanoporous Texture as Both Anode Host and Artificial SEI for Dendrite-Mitigated Lithium-Metal Batteries. J. Mat. Chem. A 9, 5606–5618. 10.1039/d0ta08782c

[B50] Xiao J. (2019). How Lithium Dendrites Form in Liquid Batteries. Science 366, 426–427. 10.1126/science.aay8672 31649185

[B51] Xu W. Wang J. Ding F. Chen X. Nasybulin E. Zhang Y. (2014). Lithium Metal Anodes for Rechargeable Batteries. Energy Environ. Sci. 7, 513–537. 10.1039/c3ee40795k

[B52] Yang C.-P. Yin Y.-X. Zhang S.-F. Li N.-W. Guo Y.-G. (2015). Accommodating Lithium into 3D Current Collectors with a Submicron Skeleton towards Long-Life Lithium Metal Anodes. Nat. Commun. 6, 8058. 10.1038/ncomms9058 26299379 PMC4560781

[B53] Yin Y.-C. Wang Q. Yang J.-T. Li F. Zhang G. Jiang C.-H. (2020). Metal Chloride Perovskite Thin Film Based Interfacial Layer for Shielding Lithium Metal from Liquid Electrolyte. Nat. Commun. 11, 1761. 10.1038/s41467-020-15643-9 32273513 PMC7145840

[B54] Yu L. Su Q. Li B. Liu W. Zhang M. Ding S. (2020). Bio-Inspired lotus Root-Like 3D Multichannel Carbon Hosts for Stable Lithium Metal Anodes. Electrochimica Acta 362, 137130. 10.1016/j.electacta.2020.137130

[B55] Zeng J. Liu Q. Jia D. Liu R. Liu S. Zheng B. (2021). A Polymer Brush-Based Robust and Flexible Single-Ion Conducting Artificial SEI Film for Fast Charging Lithium Metal Batteries. Energy Storage Mater. 41, 697–702. 10.1016/j.ensm.2021.07.002

[B56] Zhang D. Dai A. Wu M. Shen K. Xiao T. Hou G. (2019a). Lithiophilic 3D Porous CuZn Current Collector for Stable Lithium Metal Batteries. ACS Energy Lett. 5, 180–186. 10.1021/acsenergylett.9b01987

[B57] Zhang L. Zheng H. Liu B. Xie Q. Chen Q. Lin L. (2021). Homogeneous Bottom-Growth of Lithium Metal Anode Enabled by Double-Gradient Lithiophilic Skeleton. J. Energy Chem. 57, 392–400. 10.1016/j.jechem.2020.09.004

[B58] Zhang R. Chen X.-R. Chen X. Cheng X.-B. Zhang X.-Q. Yan C. (2017a). Lithiophilic Sites in Doped Graphene Guide Uniform Lithium Nucleation for Dendrite-Free Lithium Metal Anodes. Angew. Chem. Int. Ed. 56, 7764–7768. 10.1002/anie.201702099 28466583

[B59] Zhang W.-J. (2011). A Review of the Electrochemical Performance of Alloy Anodes for Lithium-Ion Batteries. J. Power Sources 196, 13–24. 10.1016/j.jpowsour.2010.07.020

[B60] Zhang X. Wang A. Lv R. Luo J. (2019b). A Corrosion-Resistant Current Collector for Lithium Metal Anodes. Energy Storage Mater. 18, 199–204. 10.1016/j.ensm.2018.09.017

[B61] Zhang Y. Liu B. Hitz E. Luo W. Yao Y. Li Y. (2017b). A Carbon-Based 3D Current Collector with Surface Protection for Li Metal Anode. Nano Res. 10, 1356–1365. 10.1007/s12274-017-1461-2

[B62] Zheng H. Zhang Q. Chen Q. Xu W. Xie Q. Cai Y. (2020). 3D Lithiophilic-Lithiophobic-Lithiophilic Dual-Gradient Porous Skeleton for Highly Stable Lithium Metal Anode. J. Mat. Chem. A 8, 313–322. 10.1039/c9ta09505e

[B63] Zhong Y. Chen Y. Cheng Y. Fan Q. Zhao H. Shao H. (2019). Li Alginate-Based Artificial SEI Layer for Stable Lithium Metal Anodes. ACS Appl. Mat. Interfaces 11, 37726–37731. 10.1021/acsami.9b12634 31549805

[B64] Zhou Z. Feng Y. Wang J. Liang B. Li Y. Song Z. (2020). A Robust, Highly Stretchable Ion-Conducive Skin for Stable Lithium Metal Batteries. Chem. Eng. J. 396, 125254. 10.1016/j.cej.2020.125254

